# Flavoured water consumption alters pharmacokinetic parameters and increases exposure of erlotinib and gefitinib in a preclinical study using Wistar rats

**DOI:** 10.7717/peerj.9881

**Published:** 2020-09-22

**Authors:** Aliyah Almomen, Hadir M. Maher, Nourah Z. Alzoman, Shereen M. Shehata, Amal Alsubaie

**Affiliations:** 1 Department of Pharmaceutical Chemistry, College of Pharmacy, King Saud University, Riyadh, Saudi Arabia; 2 Faculty of Pharmacy, Department of Pharmaceutical Analytical Chemistry, Alexandria University, Alexandria, Egypt; 3 Biological Products Evaluation Directorate, Saudi Food and Drug Authority, Riyadh, Saudi Arabia

**Keywords:** Flavored water, Tyrosin Kinase Inhibitors, Erlotinib, Gefitinib, Pharmacokinetic, Rat, Drug interaction, Toxicity, In vivo, UPLC

## Abstract

**Background:**

Erlotinib (ERL) and Gefitinib (GEF) are considered first line therapy for the management of non-small cell lung carcinoma (NSCLC). Like other tyrosine kinase inhibitors (TKIs), ERL and GEF are mainly metabolized by the cytochrome P450 (CYP450) CYP3A4 isoform and are substrates for transporter proteins with marked inter-/intra-individual pharmacokinetic (PK) variability. Therefore, ERL and GEF are candidates for drug-drug and food-drug interactions with a consequent effect on drug exposure and/or drug-related toxicities. In recent years, the consumption of flavoured water (FW) has gained in popularity. Among multiple ingredients, fruit extracts, which might constitute bioactive flavonoids, can possess an inhibitory effect on the CYP450 enzymes or transporter proteins. Therefore, in this study we investigated the effects of different types of FW on the PK parameters of ERL and GEF in Wistar rats.

**Methods:**

ERL and GEF PK parameters in different groups of rats after four weeks consumption of different flavours of FW, namely berry, peach, lime, and pineapple, were determined from plasma drug concentrations using ultra-performance liquid chromatography–tandem mass spectrometry (UPLC-MS/MS).

**Results:**

Data indicated that tested FWs altered the PK parameters of both ERL and GEF differently. Lime water had the highest impact on most of ERL and GEF PK parameters, with a significant increase in C_max_ (95% for ERL, 58% for GEF), AUC_0–48_ (111% for ERL, 203% for GEF), and AUC_0–∞_ (200% for ERL, 203% for GEF), along with a significant decrease in the apparent oral clearance of both drugs (65% for ERL, 67% for GEF). The order by which FW affected the PK parameters for ERL and GEF was as follows: lime > pineapple > berry > peach.

**Conclusion:**

The present study indicates that drinking FW could be of significance in rats receiving ERL or GEF. Our results indicate that the alteration in PKs was mostly recorded with lime, resulting in an enhanced bioavailability, and reduced apparent oral clearance of the drugs. Peach FW had a minimum effect on the PK parameters of ERL and no significant effect on GEF PKs. Accordingly, it might be of clinical importance to evaluate the PK parameters of ERL and GEF in human subjects who consume FW while receiving therapy.

## Introduction

Lung cancer is one of the leading causes of death among men and women worldwide, with the difficult to treat non-small cell lung carcinoma (NSCLC) accounting for up to 75% of lung cancer cases (Torre, Siegel & Jemal, 2016; Bethune et al., 2010). Since the discovery of the activating mutation in the epidermal growth factor receptors (EGFR) kinase domain in a subset of patients with NSCLC, accounting for 17% of cases in North America and Europe and 50% of cases in Asia, EGFR tyrosine kinase inhibitors (TKIs) were perceived as an attractive treatment intervention with a 65–90% response rate  (Brehmer et al., 2005; Ladanyi & Pao, 2008). When compared to standard chemotherapeutic platinum-based treatments of advanced NSCLC, first generation TKIs, erlotinib (ERL) and gefitinib (GEF), showed superior response rates and progression-free survival rates (Sullivan & Planchard, 2016).

ERL and GEF, so called adenosine triphosphate (ATP)-competitive EGFR TKIs, are orally administered TKIs that act by competing with ATP on the EGFR intracellular ATP binding pockets resulting in an inhibition of cell proliferation, angiogenesis, and metastatic potential (Brehmer et al., 2005; Bronte et al., 2014). After oral administration, ERL is rapidly absorbed from the gastrointestinal system with a time to reach maximum concentration (*t*_max_) of 1.4 h in healthy subjects and a mean bioavailability (F) of 59% (Scheffler et al., 2011). In cancer patients, ERL’s *t*_max_ and F are 3 h and 76%, respectively (Scheffler et al., 2011). On the other hand, GEF is moderately absorbed after oral administration with *t*_max_ of 3 h and median F of 57% in healthy subjects (Scheffler et al., 2011). In cancer patients, however, GEF’s *t*_max_ is about 7 h and median F is around 59% (Scheffler et al., 2011). ERL and GEF each undergo extensive tissue distribution with relatively long half-lives (*t*_1∕2_). The *t*_1∕2_ of ERL is about 24.4 h in healthy patients and up to 40.9 h in cancer patients, while GEF’s *t*_1∕2_ is about 41 h in healthy patients and 48 h in cancer patients (Scheffler et al., 2011; Lu et al., 2006).

ERL and GEF are substrates of the ATP-binding cassette (ABC) transporters including P-glycoprotein (P-gp), breast cancer resistance protein (BCRP), and organic anion-transporting polypeptides (OATPs), particularly the OATP2B1 (Scheffler et al., 2011; Johnston et al., 2014; Marchetti et al., 2008; Agarwal et al., 2010; Bauer et al., 2018; Chen et al., 2011). Factors that affect the expression or action of these transporters can have PK implications on drugs that are substrates to these transporters  (Marchetti et al., 2008). Transporter inhibitors were used as a strategy to overcome TKIs drug resistance where significant increase in drug exposure was found  (Marchetti et al., 2008; Beretta et al., 2017). TKIs are mainly metabolized in the liver with reported PK variability, which can be attributed to differences in profiles of the liver metabolizing enzymes, namely CYP450s (Scheffler et al., 2011; Li et al., 2007). It can, therefore, be inferred that factors altering CYP450 enzymes or ABC transporters such as disease states, drugs (drug-drug interactions), or food (food-drugs interactions), should be considered when prescribing TKIs.

Drug-food interactions can result from the interaction of drugs with some natural constituents such as alkaloids and flavonoids, as well as other artificial components which are present in daily consumed food and drinks (Bansal et al., 2009; Li et al., 2016; Li, Revalde & Paxton, 2017; Abdallah et al., 2015; Kamenickova & Dvorak, 2012). Alteration in CYP enzymes and ABC can consequently alter the PK parameters of their substrates, i.e., bioavailability, tissue penetration, biliary excretion with a possible occurrence of drug-related toxicities (Bansal et al., 2009; Li et al., 2016; Li, Revalde & Paxton, 2017; Abdallah et al., 2015). Fruits and vegetables are known to be rich sources of bioactive flavonoids which can have different inhibitory effects on CYP450 metabolizing enzymes such as CYP3A4 and transporter proteins including P-gp  (Bansal et al., 2009; Li et al., 2016; Li, Revalde & Paxton, 2017; Abdallah et al., 2015). The inhibition of P-gp by flavonoids can be comparable to that by verapamil and cyclosporine, two very well-known P-gp inhibitors (Bansal et al., 2009). For example, flavonoids, such as quercetin, increases the maximum serum concertation (C_max_) and prolong the *t*_1∕2_ of paclitaxel (Choi, Jo & Kim, 2004). In addition to quercetin, other flavonoids such as kaempferol was able to increase tamoxifen C_max_ in female rats and both C_max_ and F in male rats  (Piao, Shin & Choi, 2008; Shin, Choi & Li, 2006).

A well-known drug-food or beverage interaction is found with the co-administration of ERL or GEF and grapefruit, among other fruits  (Mouly et al., 2017). Grapefruit, pomegranate, star fruit, and Seville oranges are known CYP450 inhibitors (Mouly et al., 2017; Molden & Spigset, 2007; Hidaka et al., 2004). They are inhibitors of the enzyme CYP3A4, a main ERL and GEF metabolizing enzyme (Li et al., 2007; Mouly et al., 2017; Molden & Spigset, 2007; Hidaka et al., 2004). It is thought that this action is mediated through a spiro-ortho-ester component, BAS 100, which is a potent CYP34A inhibitor (Molden & Spigset, 2007; Hidaka et al., 2004; Smith et al., 2008). Other juices such as orange, grapefruit, and apples are known to inhibit the activity of the influx transporters expressed in enterocytes and hepatocytes (OATP 1A2, 1B1, and 2B1), which can cause a drastic decrease in substrate drugs plasma levels (Mouly et al., 2017). Thus, it is advisable to stop consuming any of the above mentioned fruits or their products while on ERL or GEF therapy (Li et al., 2007; Collado-Borrell et al., 2016). Similar food/beverage drug interaction were reported with the coadministration of green tea and ERL, as well as the Chinese medicinal plant (*Marsdenia tenacissima*) and GEF (Han et al., 2014; Maher et al., 2017).

Flavoured bottled water (FW), carbonated (sparkling) and still, are gaining popularity among consumers in recent years (Lorenc et al., 2019; Bilek et al., 2014; Brown et al., 2007). It was reported that retail sales of FW have increased by 72% in 2018 (Purdy, 2019). FW is water mixed with natural or artificial flavouring agents (Bilek et al., 2014; Brown et al., 2007). In conjunction with flavouring agents, still FW can contain sweetening agents like aspartame, antioxidant agents like ascorbic acid, acidifying agents like citric acid, and preservatives like sodium benzoate (Bilek et al., 2014). Compared to still FW, carbon dioxide and sodium bicarbonate are extra components found in carbonated FW (Bilek et al., 2014; Cuomo et al., 2009). However, carbon dioxide is mainly lost in the air once the bottle of carbonated FW is opened and a very small amount reaches the stomach upon consumption (Cuomo et al., 2009). A study by Kamenickova & Dvorak (2012) indicated that FW can contain fruits or herbal extracts among other ingredients which may influence the transcriptional activity of CYP enzymes. Also, FW which contains lemon, green tea, ginseng, guava, or passion fruit components may cause the activation of CYP1A2, increasing the chances of food-drug interactions (Kamenickova & Dvorak, 2012).

Although some argue that extrapolating the results of experiments on animal’s PK to that of human’s PK is not always appropriate due to the differences in some CYP enzyme isoforms, Matsubara et al. (2004), indicated that similarities between human and rat can exist, like the similarity found between human CYP3A4 and rat CYP3A62 isoform. Although the activity of multidrug resistance-associated protein (MRP) in rat hepatocytes showed higher activity than human hepatocytes, humans hepatocytes exhibited higher BCRP activity than rat hepatocytes (Li et al., 2008). However, no species difference in the activity P-gp was found between rat and human hepatocytes (Li et al., 2008).

Therefore, in this study we aimed to investigate preclinically the effect of different flavours of still FW on the PK parameters of ERL and GEF, in Wistar rat.

## Materials & Methods

### Experimental animals

Fifty healthy male Wistar rats weighing 250  ± 30 g were obtained from the animal house, College of Pharmacy, King Saud University, Riyadh, Saudi Arabia. Rats were randomly divided into ten cages (*n* = 5). All individualized ventilated cages (IVC) type IV used in this study were of 20″L × 11″W × 9″ in dimension capable of hosting 4–5 rats. Cages density, bedding, and sanitation frequency was similar in all cages. Cages were housed at room temperature (25 °C) and at an average relative humidity of 50%. Daily observation of all rats was required to ensure that all animals maintained good health. All animal experiments strictly followed the guidelines of the Ethical Committee for Performing Studies on Animals, King Saud University, Riyadh, Saudi Arabia, protocol number KSU-SE-19-13.

### Study design

Four weeks before drug administration, cages were divided into five groups, two cages per group (*n* = 10), and rats had free access to commercial standard food and either water (group I, control group) or one of the FW (tested groups). Four types of FW, (Aquafina®, a product of Pepsi-Cola®, Saudi Industrial Projects Company, Jeddah, Saudi Arabia) with different flavours, specifically berry (group II), peach (group III), lime (group IV), and pineapple FW (group V) were used. Other components found in FW used in this study include water, preservatives (sodium phosphate, potassium sorbet, sodium benzoate), acidifiers (citric acid, maleic acid), sweeteners (aspartame, potassium acesulfame, allose), acidity regulator (sodium citrate), and antioxidant agent (calcium disodium EDTA). An estimation of the average amount of water and FW consumed by rats in each treatment group is depicted in Table S1. The parallel design was used in this study to avoid animals’ physiological changes that could occur in the cross-over design, possibly affecting PK profiles of drug substances. All animals involved in the study were deprived from food, but not water/FW, for at least 12 h before drug administration. Water and FW groups were then randomly subdivided (*n* = 5/each), where one cage of water or FW received one dose of ERL (20 mg/kg), and the other cage received GEF (20 mg/kg) suspended in 0.5% carboxymethyl cellulose through oral gavage; ERL and GEF were obtained from Pfizer Inc. (New York, USA). Drugs were administered to the rats between 7:00 am and 8:00 am. Rats were separately weighed from day one of water/FW consumption prior to drug administration for accurate dose calculation (Fig. S1). Blood samples (0.3 mL) were collected from each rat immediately prior to dosing (0 time) and at predetermined time points (0.5, 1, 2, 3, 5, 24, and 48 h) post drug administration. Blood samples were withdrawn and processed as described previously (Maher et al., 2017; Maher, Alzoman & Shehata, 2016; Maher, Alzoman & Shehata, 2018). Specifically, blood samples were withdrawn from the retro-orbital sinus into a series of heparinized tubes. Plasma was separated from collected blood samples by centrifugation at 4,500 rpm for 30 min where the centrifuge was maintained at 4 °C, and then stored at −20 °C until the day of analysis. Plasma samples were diluted as described previously, then ERL and GEF concentrations were determined from plasma samples using UPLC-MS/MS system, Waters Xevo TQ-S (Waters, Singapore), equipped with Acquity UPLC C 18 column (100 × 1.0 mm, 1.7 µm particle size) (Waters, Dublin, Ireland) (Maher, Alzoman & Shehata, 2016). A mixture of acetonitrile: water (80: 20, v/v), with 0.1% formic acid was used as a mobile phase at a flow rate of 0.2 mL/min (Maher, Alzoman & Shehata, 2016). Quantitative determination of ERL or GEF was conducted using a linear calibration curve between the range of 0.025-100 ng/mL with a correlation coefficient of 0.99. Quantitation of the analytes was performed using the multiple reaction monitoring (MRM) with positive ionization at m/z 447.25 > 128.08 (GEF), m/z 394.20 > 278.04 (ERL), and m/z 426.26 > 175.07 for the internal standard domperidone (DOM). The carry-over effect commonly encountered during the UPLC-MS/MS analysis, previously evaluated as an important validation parameter during method development and validation, did not exceed 15% for concentrations above lower limit of quantification (LLOQ) and 20% for concentrations at LLOQ level.

**Figure 1:**
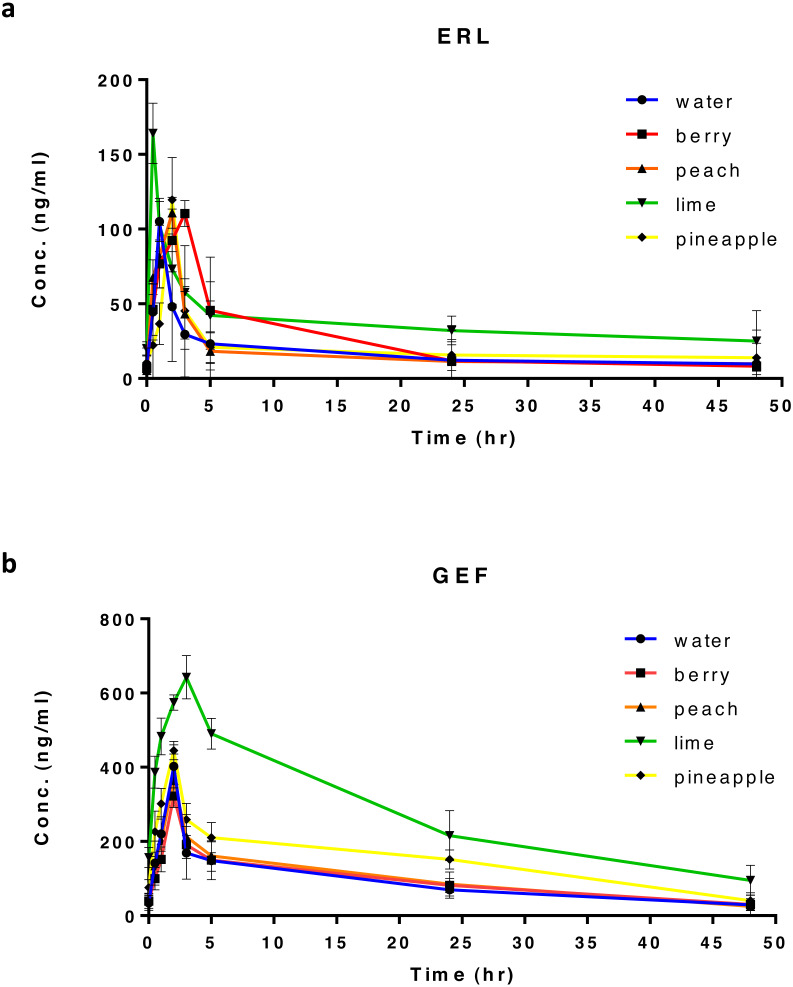
Plasma concentration-time profile after oral administration of 20 mg/kg of ERL (A), or GEF (B) in rats, along with different types of FW (*n* = 5) per group. Suitable dilutions of plasma samples were made before actual analysis.

### PK analysis

In all treated rats, ERL and GEF PK analyses were performed using non-compartmental analysis (NCA) with the aid of Excel 2010, PKSolver Add-In. C_max_ and *t*_max_ were obtained from the corresponding plasma concentration–time curve (Figs. 1A and 1B). Linear regression of the terminal phase of log-linear plasma concentration–time curve, using three points for ERL and four points for GEF (Figs. S2A and S2B), was used to estimate the terminal elimination rate constant (*λ*_*z*_) and calculate *t*_1∕2_ from the formula, *t*_1∕2_ = 0.693∕*λ*_*z*_. The area under the plasma concentration–time curve from time 0 to the last sampling time *t*, 48 h (AUC_0–*t*_) was calculated using the trapezoidal rule. The apparent oral clearance (CL/F) was calculated as followed: CL/F = dose/AUC_0–∞_, where CL was drug clearance, F was drug bioavailability, dose was 20 mg/kg for ERL or GEF, and AUC_0–∞_ is the area under the plasma concentration–time curve from time 0 to ∞. AUC_0–∞_ was also derived by summing up AUC_0–*t*_ andthe area obtained by extrapolation from time t to ∞. The latter area was calculated from the division of the last measured concentration (C_last_) by *λ*_*z*_. The ranking by which FW was considered to have the highest to the lowest impact on ERL or GEF PK parameters is based on the number of PK parameters affected by the coadministration of FW with each drug.

### Statistical analysis

Results were presented as mean ± SD. PK parameters of all treated groups following the consumption of FW were compared with control (rats having free access to water), using one-way ANOVA and Bonferroni’s multiple comparisons test. Statistical significance was obtained with *p*-values ≤ 0.05.

## Results

### Effect of FW on the PKs of ERL

**Figure 2:**
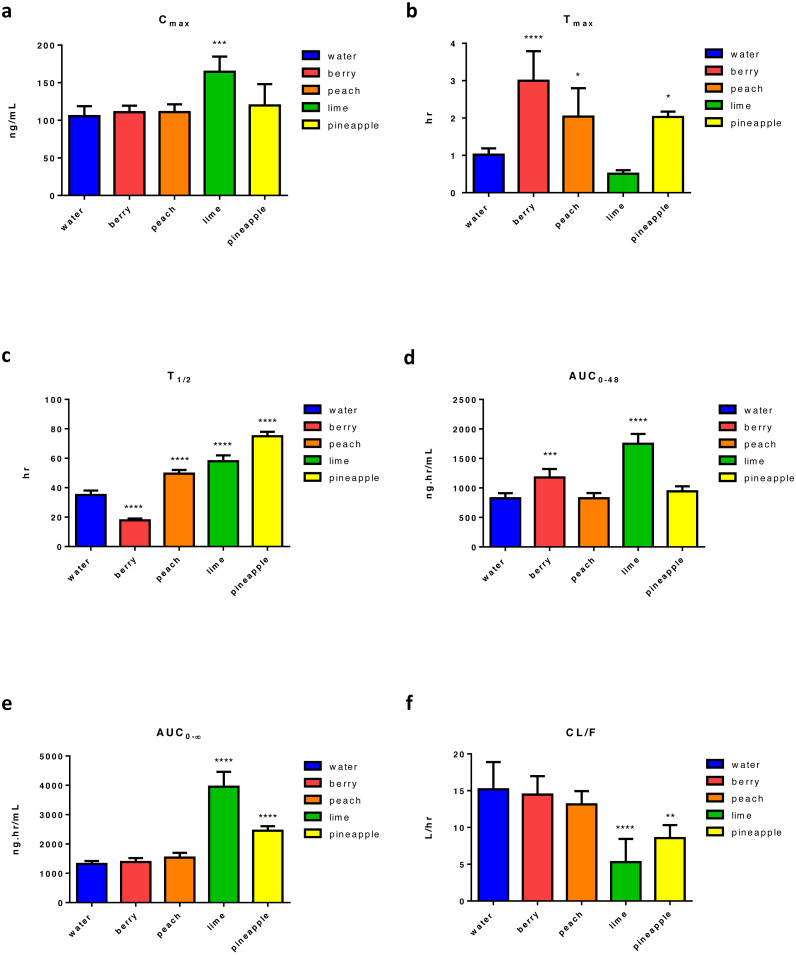
Main pharmacokinetic parameters of ERL following four weeks administration of different types of FW in rats relative to control (*n* = 5) per group. Statistical significance was obtained with *p*-values ≤ 0.05, where * *P* ≤ 0.05, ** *P* ≤ 0.01, *** *P* ≤ 0.001, and **** *P* < 0.0001.

Following four weeks of FW consumption, results show that lime FW was the only FW causing a significant increase in ERL’s C_max_ (95% increase) (Fig. 2A). On the other hand, a significant increase in *t*_max_ was found with almost all tested FW, showing three times increase with berry FW and two times increase with peach and pineapple. The only decrease in *t*_max_ was found with lime FW, which was not statistically significant (Fig. 2B). Also, a significant change in *t*_1∕2_ with almost 49% decrease with berry, and about 41%, 65%, and 113.8% increase was noticed with peach, lime, and pineapple FW, respectively (Fig. 2C). While berry FW was only able to increase AUC_0–48_ by 42.35%, pineapple FW increased the AUC_0–∞_ by 86%. Lim FW, on the other hand, was able to increase both AUC_0–48_ and AUC_0–∞_, of about 111% and 200%, respectively (Figs. 2D and 2E). Regarding the apparent oral clearance, lime and pineapple FW caused a significant decrease of 65% and 30%, respectively (Fig. 2F). Results revealed that peach FW had no significant effect on other measured PK parameters including C_max_, AUC_0–48_, AUC_0–∞_, and CL/F. However, compared with other types of FW, lime FW had the most significant effect on ERL PK with the maximum increase in C_max_, AUC_0–48_ and AUC_0–∞_, along with maximum decrease in ERL apparent clearance (Table S2).

### Effect of FW on the PKs of GEF

**Figure 3:**
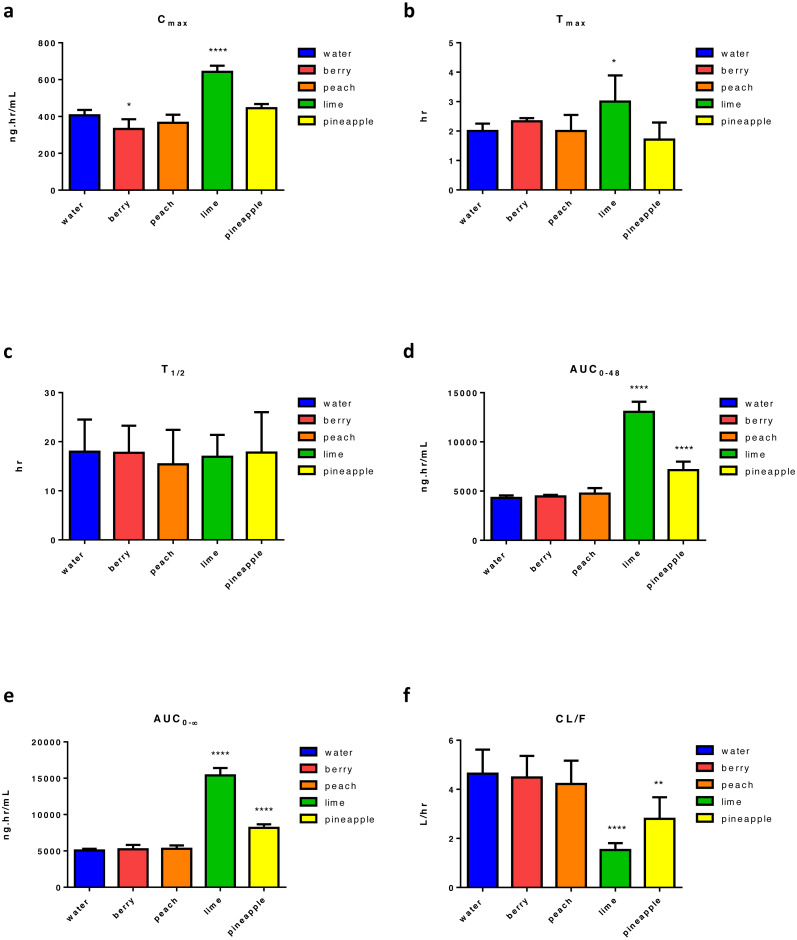
Main pharmacokinetic parameters of GEF, following four weeks administration of different types of FW in rats relative to control (*n* = 5) per group. Statistical significance was obtained with *p*-values ≤ 0.05, where * *P* ≤ 0.05, ** *P* ≤ 0.01, *** *P* ≤ 0.001, and **** *P* < 0.0001.

Four weeks of FW consumption by animals followed by GEF administration resulted in a significant change in C_max_ relative to control. There was a 58% increase in C_max_ found with lime and an 18% decrease with berry FW (Fig. 3A). Moreover, there was an almost 50% significant increase in *t*_max_ with lime (Fig. 3B). None of the tested FW caused a significant change in *t*_1∕2_ (Fig. 3C). Two types of FW, lime and pineapple, had significantly affected GEF apparent bioavailability and increases in AUC_0–48_ andAUC_0–∞_ were recorded. Consequently, a significant reduction in CL/F occurred. As with ERL, the maximal effect on AUC_0–∞_ and was found with lime FW with almost 203% increase in both AUCs (Figs. 3D and 3E) followed by pineapple FW with almost 65% and 61% increase in AUC_0–48_ and AUC_0–∞_, respectively. Thus, a significant reduction of the apparent clearance of almost 67% and 40% of GEF was recorded with lime and pineapple FW, respectively (Fig. 3F). Table S3 summarizes the main PK parameters of GEF following the intake of FW.

## Discussion

In this study, we investigated the effect of different flavours of still FW on the PKs of ERL and GEF in male Wistar rats. Like other TKIs, ERL and GEF are susceptible to CYP3A4-mediated metabolism and are actively transported by transporters like P-gp, BCRP, OATPs  (Scheffler et al., 2011; Johnston et al., 2014; Marchetti et al., 2008; Agarwal et al., 2010; Bauer et al., 2018; Chen et al., 2011). Thus, altering CYP enzymatic activity and/or protein transporters could significantly affect ERL or GEF exposure with consequent impact on therapeutic efficacy and/or drug-related toxicities.

A previous study suggests a possible gender specific differences in PK parameters between male and female rats knowing that CYP2C11, CYP2C13, and CYP3A2 are expressed in male rats whereas CYP2C12 is expressed in female rats, which can cause a difference in drug metabolism (Czerniak, 2001). Moreover, female rat oestrus cycle can have an effect on liver metabolic enzyme, thus male rats were chosen in this study to eliminate any other possible variabilities that can effect ERL and GEF PKs (Kulkarni et al., 2012).

Tested FW altered most of ERL and GEF measured PK parameters, and the impact was in the following order: lime > pineapple > berry > peach for both ERL and GEF. Tables S2 and S3 revealed that peach FW had the least effect on ERL and no significant effect on GEF PKs. Berry FW significantly affected the exposure (AUC_0–48_) of ERL, but no effect was found with GEF. Despite that both pineapple and lime FW had a significant effect on most ERL and GEF measured PK parameters, lime FW increased drug exposure (increased C_max_, AUC_0–∞_) and decreased CL/F more extensively than pineapple FW.

A literature review indicated that the concomitant consumption of fruits/vegetables can influence the PK parameters of some drugs, and hence highlights the problem of drug-juice interactions (Sasaki et al., 2017; Mallhi et al., 2015). This influence can be mainly attributed to the potential inhibitory effect of juice components on CYP metabolizing enzymes, drug transporters including P-gp, and OATPs (Sasaki et al., 2017; Mallhi et al., 2015). It was reported previously that lime juice has a similar effect on drugs to grapefruit juice due to the similarly in the polyphenol and bergamottin contents which can act as inhibitors to the CYP3A4 and CYP2C9 enzymes (Karmakar et al., 2015; Bailey, Dresser & Bend, 2003; Steuck, Hellhake & Schebb, 2016; Hanley et al., 2011). CYP3A4 and P-gp, which is usually present in the GI system and the liver, have overlapping substrates as well as inhibitors inferring that P-gp might also be inhibited by grapefruit juice (Bailey, 2010). Accordingly, lime juice may have similar drug interactions to those reported with grapefruit, i.e., increase in the bioavailability of co-administered medications (Dahan & Altman, 2004; Hollander et al., 1995). In this respect, it is noteworthy to mention that the bioavailability of dasatinib’s, a known TKI, usually increases following the ingestion of grapefruit juice (Fleisher et al., 2015). Thus, the lime FW-induced increase in the bioavailability of ERL/GEF could be attributed to the inhibition of CYP3A4-mediated metabolism and/or transporter proteins.

Pineapple juice also has an inhibitory effect on the activity of CYP2C9 enzyme due to its bromelain content (Hidaka et al., 2008). Some of the reported pineapple juice-drug interactions were found with diclofenac, warfarin, and tolbutamide, all of which are CYP2C9 substrates (Hidaka et al., 2008). Although pineapple has no reported modulatory effect on CYP3A4 enzymes, pineapple-induced increases in ERL/GEF bioavailability could be attributed to the mucus disrupting property of bromelain. Being a proteolytic enzyme, it could increase the muco-penetration and diffusion of drugs through the mucous barriers, increasing the systematic exposure (Murgia et al., 2018). Also, a study by Amadi et al., indicates that pineapple can possibly have an inhibitory effect on P-gp and OATP transporters, and thus can increase the exposure of drugs that are substrates to these transporters such as fexofenadine (Amadi & Barileela, 2018). While P-gp found on the intestinal epithelium act as efflux proteins, decreasing the bioavailability of drugs substrate, OATP are influx proteins that can influence the absorption of drug substrates and increase bioavailability (Amadi & Barileela, 2018). The increase in ERL and GEF exposure in our study indicates that pineapple juice might have a favourable inhibition of P-gp over OATP (Amadi & Barileela, 2018).

The effect of different types of berry juice on drug metabolizing enzymes was also studied previously (Mallhi et al., 2015). It was reported that cranberry juice can inhibit CYP3A4 or CYP2C9 (Mallhi et al., 2015; Uesawa & Mohri, 2006; Ushijima et al., 2009). However, studies indicated that it is unlikely that cranberry juice can cause a significant drug interactions with drug metabolized by CYP3A4 or CYP2C9  (Wanwimolruk et al., 2012; Pham & Pham, 2007). Other berries such as black raspberry and blueberry are reported to be weak inhibitors of CYP3A1 and CYP3A4, respectively  (Mallhi et al., 2015; Dreiseitel et al., 2008). This might justify the different effect berry FW has on ERL’s exposure as compared to lime FW, and the negligible effect on GEF PKs except for the C_max_. This may also be the case for peach FW, where a previous report has only described the down-regulatory effect of peach supplementation on CYP2A and CYP2b1/2, but not on CYP3A4 (Canistro et al., 2016).

Although it is possible that the dose of flavouring agent used could have influenced the PK parameters of ERL and GEF differently, Table S1 shows that lime and pineapple FW were consumed less than berry and peach, probably due to the sour or bitter flavours of lime and pineapple compared to berry and peach. Yet, lime and pineapple had more impact on the PK of both drugs. Also, the literature indicates that weight or obesity can affect the PK parameters of drugs due to an alteration in CL and volume of distribution (*V*_*d*_), and therefore *t*_1∕2_ (Hanley, Abernethy & Greenblatt, 2010). In our study, however, there was no significant difference in weights among rats in different FW groups ruling out the possible impact of the rats’ weight on ERL and GEF PKs (Fig. S1).

Artificial sweetening agents such as aspartame, are another component of FW. To our knowledge, there was no reported drug interaction between aspartame and ERL or GEF. Also, other studies indicate that aspartame has no effect on hepatic CYP enzymes such CYP1A2, an enzyme involved in ERL metabolism, or CYP2A3 (Li et al., 2007; Kamenickova et al., 2013; Vences-Mejia et al., 2006). Thus, aspartame might not be involved in the metabolism of ERL and GEF. Further evaluation of aspartame’s effect on ERL/GEF metabolism is needed.

ERL and GEF are weak bases that are better absorbed in an acidic pH, and thus alteration of gastric pH can affect their absorption rate  (Kumarakulasinghe et al., 2016). Acidifiers and acid regulators are components of FW. A study by van Leeuwen et al., indicated that the concomitant use of cola and acidic beverage can significantly increase ERL mean drug exposure. However, the effect on patients were marginal and clinically irrelevant  (Van Leeuwen et al., 2016). Also, Fotaki and Klein showed that carbonated beverages can change the stomach pH which in turn can affect drugs absorption, dissolution, and stomach emptying (Fotaki & Klein, 2013). Therefore, we cannot rule out the possible effect of acidifiers on ERL or GEF PK. In regards to the antioxidant EDTA, it was reported previously that it can prolonged gastric emptying and can act as an inhibitor of gastric acid, which could reduce drug exposure (Shafer et al., 1985). Such an effect was not observed in here, which can imply that their relatively low concentration in FW makes them not capable of inducing such an effect. Regarding preservatives such as sodium phosphate and potassium sorbate there were no reports to our knowledge that evaluated their effects on drug absorption or metabolism. Thus, further studies in this area are needed.

It is noteworthy to mention that the blood sampling time (up to 48 h) in this study was adequate to carry out a fair comparison of the overall-plasma concentration–time profile among different rat groups, which was the main purpose of our study. However, for more accurate estimation of the elimination phase, the total sampling time should have been further extended, particularly for ERL.

## Conclusion

In conclusion, the present study indicates that drinking FW could be of significance in rats receiving ERL or GEF. Furthermore, our results indicate that lime FW had the highest impact on ERL and GEF PKs in terms of the number of PK parameters altered (C_max_, *t*_max_, *t*_1∕2_, AUC_0–48_, AUC_0–∞_ and CL/F). The order by which the FW effected the PK parameters were as follows: lime > pineapple > berry > peach for both ERL and GEF. Peach had nearly negligible effect on ERL and no effect on GEF PKs. Therefore, it might be of clinical importance to evaluate the PK parameters of ERL and GEF in human subjects who consume FW while on therapy.

##  Supplemental Information

10.7717/peerj.9881/supp-1 Table S1 Estimated amount of water and FW consumed by rats in the four-week period prior to ERL or GEF administration ^a^

10.7717/peerj.9881/supp-2 Figure S2 Log plasma concentration-time profile after the oral administration of 20 mg/kg of ERL or GEFin rats, along with different types of FW (*n* = 5) Suitable dilutions of plasma samples were made before actual analysis.

10.7717/peerj.9881/supp-3 Figure S1 Average rat weight for ERL (a) and GEF (b) groups (*n* = 5)

10.7717/peerj.9881/supp-4 Table S2 Main pharmacokinetic parameters of ERL following four weeks administration of different types of FW in rats in comparison to control (*n*= 5)

10.7717/peerj.9881/supp-5 Supplemental Information 5 ERL PK stats

10.7717/peerj.9881/supp-6 Supplemental Information 6 GEF stats

10.7717/peerj.9881/supp-7 Table S3 Main pharmacokinetic parameters of GEF following four weeks administration of different types of FW in rats in comparison to control (*n*= 5)

10.7717/peerj.9881/supp-8 Supplemental Information 8 Raw data

10.7717/peerj.9881/supp-9 Supplemental Information 9 Weight change stats group 1 and group 2

10.7717/peerj.9881/supp-10 Supplemental Information 10 Data in PKSolver

## Additional Information and Declarations

### Data Availability

The following information was supplied regarding data availability: Raw data is available as Supplemental Files.
