# Supplementary material for: Flavoured water consumption alters pharmacokinetic parameters and increases exposure of erlotinib and gefitinib in a preclinical study using Wistar rats
Source: PeerJ. 2020 Sep 22;8:e9881. doi: 10.7717/peerj.9881 (PMC7518156; doi:10.7717/peerj.9881)
Supplement: Table S1 [file peerj-08-9881-s001.docx]

**Table S.1** Estimated amount of water and FW consumed by rats in the four-week period prior to ERL or GEF administration ^a^.

| **Group** | **ml/ week** |
| --- | --- |
| Group I (water) | 14.28 |
| Group II (berry FW) | 71.42 |
| Group III (peach FW) | 71.42 |
| Group IV (lime FW) | 57.14 |
| Group V (pineapple FW) | 28.57 |

^a^ Amounts were estimated by dividing the amount of consumed water by the number of rats in each cage.
