# Supplementary material for: Flavoured water consumption alters pharmacokinetic parameters and increases exposure of erlotinib and gefitinib in a preclinical study using Wistar rats
Source: PeerJ. 2020 Sep 22;8:e9881. doi: 10.7717/peerj.9881 (PMC7518156; doi:10.7717/peerj.9881)
Supplement: Figure S2 — Suitable dilutions of plasma samples were made before actual analysis. [file peerj-08-9881-s002.pdf]

a

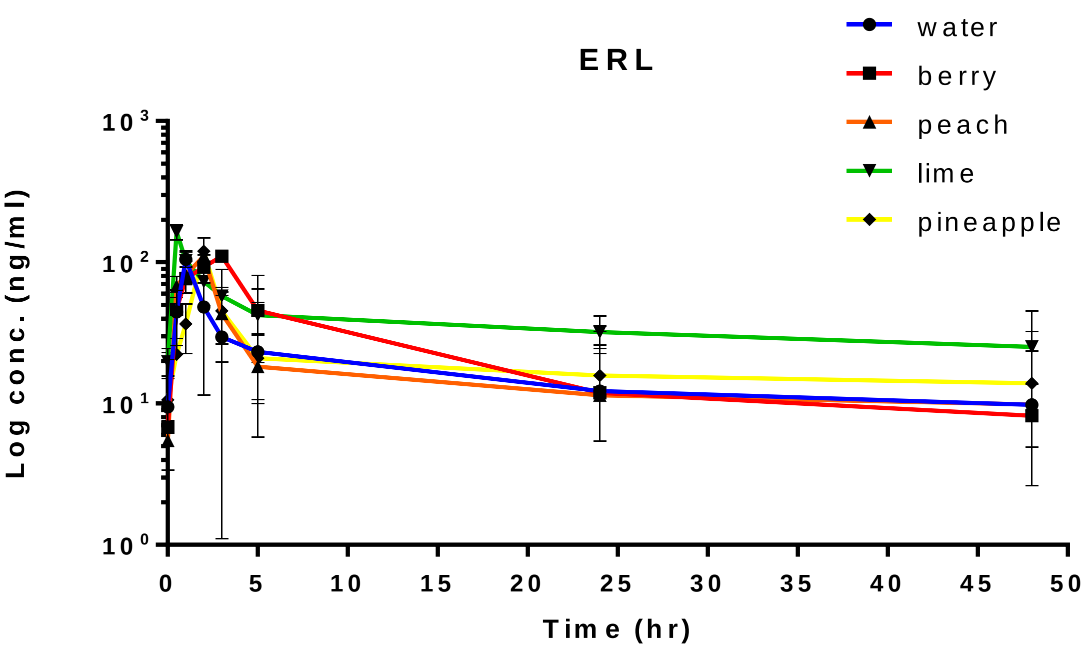
ERL
water
berry
peach
lime
pineapple
Log conc. (ng/ml)
10 3
10 2
10 1
10 0
0
5
10
15
20
25
30
35
40
45
50
Time (hr)

b

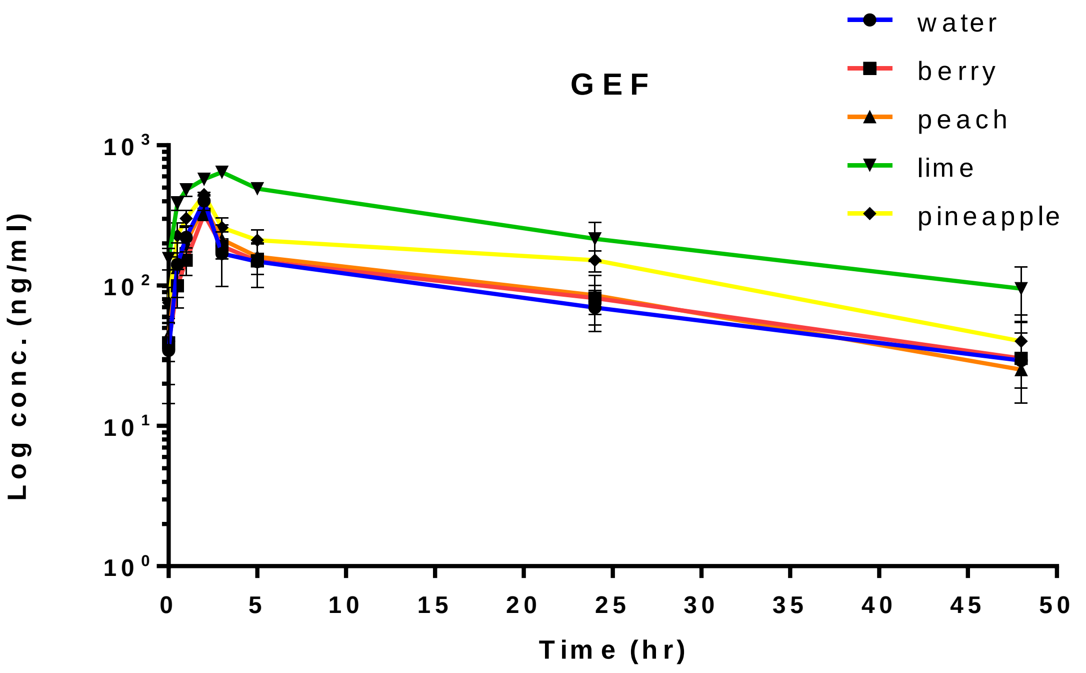
GEF
water
berry
peach
lime
pineapple
Log conc. (ng/ml)
10 3
10 2
10 1
10 0
0
5
10
15
20
25
30
35
40
45
50
Time (hr)

Figure.S2 Log plasma concentration-time profile after an oral administration of 20 mg/kg of ERL (a), or GEF (b) in rats, along with different types of FW (n=5). Suitable dilutions of plasma samples were made before actual analysis.
