# Supplementary figures and images for: Flavoured water consumption alters pharmacokinetic parameters and increases exposure of erlotinib and gefitinib in a preclinical study using Wistar rats
Source: PeerJ. 2020 Sep 22;8:e9881. doi: 10.7717/peerj.9881 (PMC7518156; doi:10.7717/peerj.9881)

**a**

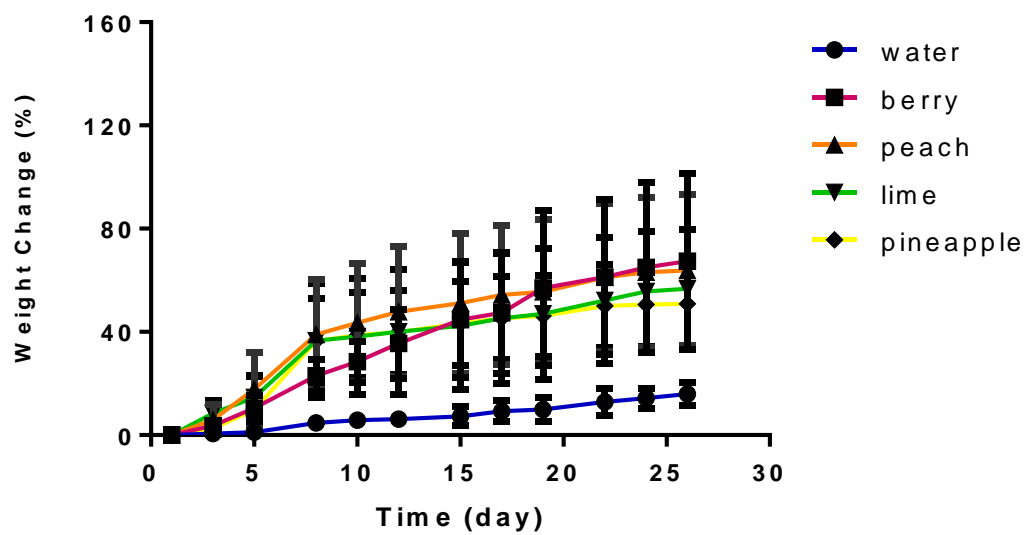

**b**

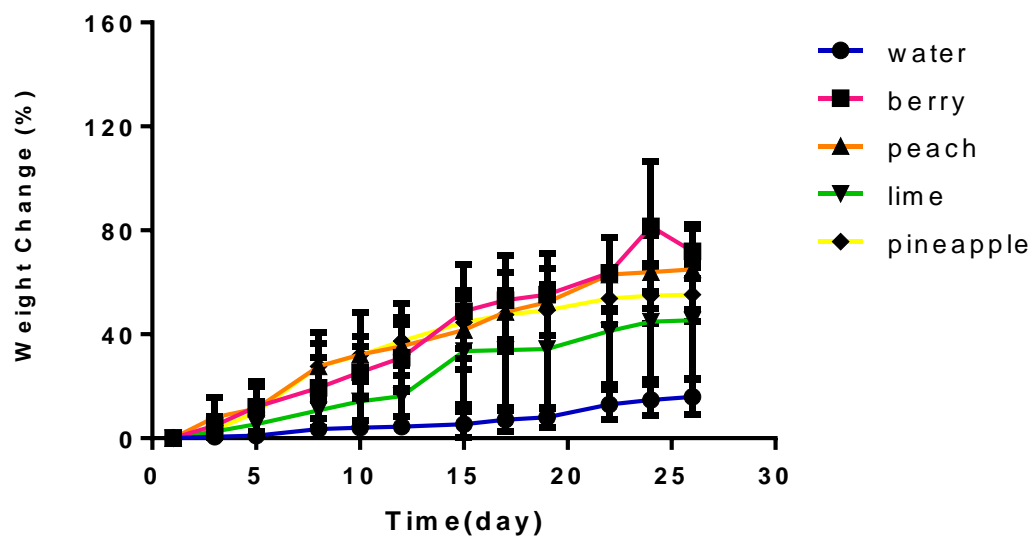

**Figure.S1** Average rat weight for ERL (a) and GEF (b) groups (n=5).

Supplement: Figure S1 [file peerj-08-9881-s003.pdf]
