# Supplementary material for: Flavoured water consumption alters pharmacokinetic parameters and increases exposure of erlotinib and gefitinib in a preclinical study using Wistar rats
Source: PeerJ. 2020 Sep 22;8:e9881. doi: 10.7717/peerj.9881 (PMC7518156; doi:10.7717/peerj.9881)
Supplement: Table S2 [file peerj-08-9881-s004.docx]

| **ERL** | | | | | | |
| --- | --- | --- | --- | --- | --- | --- |
|  | **Flavor** | **Mean (SD)** | **95% CI of diff.** | **F-ratio** | **(DFn, DFd)** | **p-value** |
| **C_max_**  **(ng/mL)** | Water  Berry  Peach  Lime  Pineapple | 105.53 (13.2)  110.81 (8.59)  110.92 10.3)  164.63***** (20.14)  119.80 (28.28) | -   \| -35.98 to 25.42 \| \| --- \| \| -36.09 to 25.31 \| \| -89.80 to -28.40 \| \| -44.97 to 16.43 \| | 9.356 | (4, 20) | 0.0002 |
| **t_max_**  **(h)** | Water  Berry  Peach  Lime  Pineapple | 1.02 (0.17)  3.00* (0.79)  2.04* (0.76)  0.51 (0.09)  2.03* (0.14) | -  -2.85-1.109  -1.891-0.1493  -0.3607-1.381  -1.881-0.1391 | 18.83 | (4, 20) | < 0.0001 |
| **t_½_**  **(h)** | Water  Berry  Peach  Lime  Pineapple | 35.06 (3.00)  17.76* (1.25)  49.58* (2.50)  57.96* (4.00)  74.96* (3.00) | -   \| 12.28 to 22.32 \| \| --- \| \| -19.54 to -9.501 \| \| -27.92 to -17.88 \| \| -44.92 to -34.88 \| | 284.9 | (4, 20) | < 0.0001 |
| **AUC_0-48_ (ng.h/mL)** | Water  Berry  Peach  Lime  Pineapple | 826.94 (85.41)  1177.21* (144.09)  826.99 (85.57)  1748.66* (167.63)  941.71 (85.57) | -   \| -556.8 to -143.7 \| \| --- \| \| -206.6 to 206.5 \| \| -1128 to -715.2 \| \| -321.3 to 91.77 \| | 53.03 | (4, 20) | < 0.0001 |
| **AUC_0-∞_ (ng.h/mL)** | Water  Berry  Peach  Lime  Pineapple | 1319.19 (100.01)  1384.83 (135.00)  1534.76 (165.00)  3952.19* (506.81)  2454.05* (150.16) | -   \| -514.8 to 383.5 \| \| --- \| \| -664.7 to 233.6 \| \| -3082 to -2184 \| \| -1584 to -685.7 \| | 93.18 | (4, 20) | < 0.0001 |
| **CL/F**  **(L/h)** | Water  Berry  Peach  Lime  Pineapple | 15.19 (3.70)  14.48 (2.50)  13.15 (1.80)  5.29* (3.15)  8.55*(1.78) | -   \| -3.965 to 5.385 \| \| --- \| \| -2.635 to 6.715 \| \| 5.225 to 14.57 \| \| 1.965 to 11.31 \| | 12.47 | (4, 20) | < 0.0001 |

*Indicates significant difference as compared with the corresponding control (water group). (p ≤ 0.05)

Standard deviation (SD), 95% confidence interval for the difference between the two means (95% CI of diff.), degree of freedom from between the columns (DFn), and degrees of freedom from within the columns (DFd)

**Table S.2** Main pharmacokinetic parameters of ERL following four weeks administration of different types of FW in rats in comparison to control (*n* = 5).
