# Supplementary material for: Flavoured water consumption alters pharmacokinetic parameters and increases exposure of erlotinib and gefitinib in a preclinical study using Wistar rats
Source: PeerJ. 2020 Sep 22;8:e9881. doi: 10.7717/peerj.9881 (PMC7518156; doi:10.7717/peerj.9881)
Supplement: Table S3 [file peerj-08-9881-s007.docx]

| **GEF** | | | | | | |
| --- | --- | --- | --- | --- | --- | --- |
|  | **Flavor** | **Mean (SD)** | **95% CI of diff.** | **F-ratio** | **(DFn, DFd)** | **p-value** |
| **C_max_**  **(ng/mL)** | Water  Berry  Peach  Lime  Pineapple | 406.47 (29.52)  322.39*(52.55)  365.43 (44.21)  642.58*(33.20)  445.13(22.58) | -   \| 10.45 to 137.7 \| \| --- \| \| -22.59 to 104.7 \| \| -299.7 to -172.5 \| \| -102.3 to 24.97 \| | 51.49 | (4, 20) | < 0.0001 |
| **t_max_**  **(h)** | Water  Berry  Peach  Lime  Pineapple | 2.00 (0.25)  2.33 (0.11)  2.00 (0.55)  3.00* (0.89)  1.71 (0.58) | -   \| -1.282 to 0.6224 \| \| --- \| \| -0.9524 to 0.9524 \| \| -1.952 to -0.04755 \| \| -0.6624 to 1.242 \| | 4.045 | (4, 20) | 0.0144 |
| **t_½_**  **(h)** | Water  Berry  Peach  Lime  Pineapple | 17.95 (6.55)  17.73 (5.52)  15.39 (7.01)  16.94 (4.44)  17.79 (8.22) | -   \| -11.02 to 11.46 \| \| --- \| \| -8.683 to 13.80 \| \| -10.23 to 12.25 \| \| -11.08 to 11.40 \| | 0.1345 | (4, 20) | 0.9676 |
| **AUC_0-48_ (ng.h/mL)** | Water  Berry  Peach  Lime  Pineapple | 4307.15 (255.25)  4459.01 (158.98) 4738.25 (562.25)  13059.21* (1022.22)  7136.22* (862.23) | -   \| -1302 to 998.1 \| \| --- \| \| -1581 to 718.9 \| \| -9902 to -7602 \| \| -3979 to -1679 \| | 157.2 | (4, 20) | < 0.0001 |
| **AUC_0-∞_ (ng.h/mL)** | Water  Berry  Peach  Lime  Pineapple | 5063.05 (225.25)  5232.05 (589.36)  5298.52 (452.22)  15386.16* (1025.22)  8168.18* (485.22) | -   \| -1236 to 897.9 \| \| --- \| \| -1302 to 831.4 \| \| -11390 to -9256 \| \| -4172 to -2038 \| | 258.1 | (4, 20) | < 0.0001 |
| **CL/F**  **(L/h)** | Water  Berry  Peach  Lime  Pineapple | 4.64 (0.98)  4.48 (0.88)  4.22 (0.95)  1.53* (0.28)  2.80* (0.88) | -   \| -1.290 to 1.610 \| \| --- \| \| -1.030 to 1.870 \| \| 1.660 to 4.560 \| \| 0.3899 to 3.290 \| | 12.79 | (4, 20) | < 0.0001 |

*Indicates significant difference as compared with the corresponding control (water group). (p ≤ 0.05)

Standard deviation (SD), 95% confidence interval for the difference between the two means (95% CI of diff.), degree of freedom from between the columns (DFn), and degrees of freedom from within the columns (DFd)

**Table S.3** Main pharmacokinetic parameters of GEF following four weeks administration of different types of FW in rats in comparison to control (*n* = 5).
